# Impact of different degrees of left ventricular strain on left atrial mechanics in heart failure with preserved ejection fraction

**DOI:** 10.1186/s12872-022-02608-7

**Published:** 2022-04-09

**Authors:** Thammarak Songsangjinda, Rungroj Krittayaphong

**Affiliations:** Division of Cardiology, Department of Medicine, Faculty of Medicine Siriraj Hospital, Mahidol University, 2 Wanglang Road, Bangkoknoi, Bangkok, 10700 Thailand

**Keywords:** Heart failure with preserved ejection fraction, Left atrial strain, Left atrial function, Cardiac magnetic resonance, Feature tracking

## Abstract

**Background:**

Impairment of left atrial (LA) function is linked to left ventricle (LV) mechanics in patients with heart failure with preserved ejection fraction (HFpEF). In this study, we set forth to determine the difference in LA mechanics compared between HFpEF patients with different degrees of LV strains using the cardiac magnetic resonance feature tracking technique.

**Methods:**

This retrospective study enrolled 79 patients with prior heart failure event and LV ejection fraction (LVEF) ≥ 50% (HFpEF group) together with 2:1 matched controls. LV global longitudinal strain (GLS), global circumferential strain (GCS), and global radial strain (GRS); LA emptying fraction (LAEF); and, LA strains consisting of reservoir phase strain (LAS_r_), conduit phase strain (LAS_cd_), and contraction phase strain (LAS_ct_) were derived from cine images. All LA parameters were compared between HFpEF subgroups (lower and higher LV strain stratified by the median of each LV strain value) and controls.

**Results:**

A total of 237 subjects were included. HFpEF had a lower LAEF and LA strain values compared with controls. The mean GLS value was significantly different between HFpEF and controls (− 13.3 ± 3.4% *vs.* − 15.4 ± 2.2%, *p* < 0.001). HFpEF with lower GLS (value ≥  − 13.1%) had significantly impaired LA mechanical parameters compared with both HFpEF with higher GLS and controls independent of potential confounders, as follows: LAEF (38.8 ± 16.6% *vs.* 48.6 ± 15.7% and 54.2 ± 12.2%), LAS_r_ (14.6 ± 7.1% *vs.* 24.3 ± 9.6% and 26.7 ± 8.8%), and LAS_cd_ (− 6.6 ± 3.9% *vs.* − 12.9 ± 6.0% and − 14.7 ± 7.4%) (post hoc analysis of variance *p* < 0.05 for all comparisons). Similarly, HFpEF with lower GCS (value ≥  − 16.6%) or lower GRS (value < 27.9%) also had significant impairment of LAS_r_ and LAS_cd_ compared with the higher strain group and controls. Abnormal LAEF (< 50%) and abnormal LAS_r_ (< 23%) are independently associated with NYHA class ≥ II (Odds ratio [OR] 3.894 [95% CI 2.202–6.885] *p* < 0.001, adjusted OR 3.382 [1.791–6.389] *p* < 0.001 for abnormal LAEF; and OR 2.613 [1.497–4.562] *p* = 0.001, adjusted OR 2.064 [1.118–2.110] *p* = 0.021 for abnormal LAS_r_).

**Conclusions:**

Patients with HFpEF were found to have impaired LV and LA mechanics. Abnormal LA mechanics was highly prevalent in HFpEF patients with lower LV strain and significantly associated with the symptomatic status of the patients.

**Supplementary Information:**

The online version contains supplementary material available at 10.1186/s12872-022-02608-7.

## Introduction

The left atrium (LA) plays a major role in heart failure (HF) with preserved ejection fraction (HFpEF) [1, 2]. When atrial compliance is lost, progressive volume-pressure overload eventually leads to deterioration of atrial function [3, 4]. This change subsequently contributes to the disease progression of HFpEF [4, 5]. The relationship between left ventricular (LV) and LA physiology is dynamic depending on the stage of heart failure [6].

Cardiac magnetic resonance (CMR) feature tracking (CMR-FT) is an emerging technique for the evaluation of LV and LA strain that demonstrates the deformation of structures [7, 8]. The advantage of this technique arises from the use of the steady-state free precession (SSFP) sequence, which has a relatively high signal-to-noise ratio, contrast-to-noise ratio [8], and it is routinely included in the current standard CMR protocol [9]. Moreover, the LA longitudinal strain value can also be derived from the same images, which enhances the benefit of this imaging technique. Previous studies have reported a difference in LA strain among different grades of LV diastolic function [10–12], and patterns of strain impairment in different stages of HFpEF have been purposed [6]; however, differential impairment of LA mechanics influenced by impaired LV mechanics in such population is not well understood. We hypothesized that HFpEF patients would have impaired LV and LA strain compared to controls, and that HFpEF with lesser LV strain values would have more impairment of LA mechanics. The aims of this study were 1) to determine the difference in LA volume and mechanical parameters, including LA emptying fraction (LAEF), LA expansion index, and LA strain, between HFpEF and controls; and, 2) to determine the differences in LA parameters compared among HFpEF patients with different degrees of LV strain using CMR-FT.

## Methods

### Study population

**Fig. 1:**
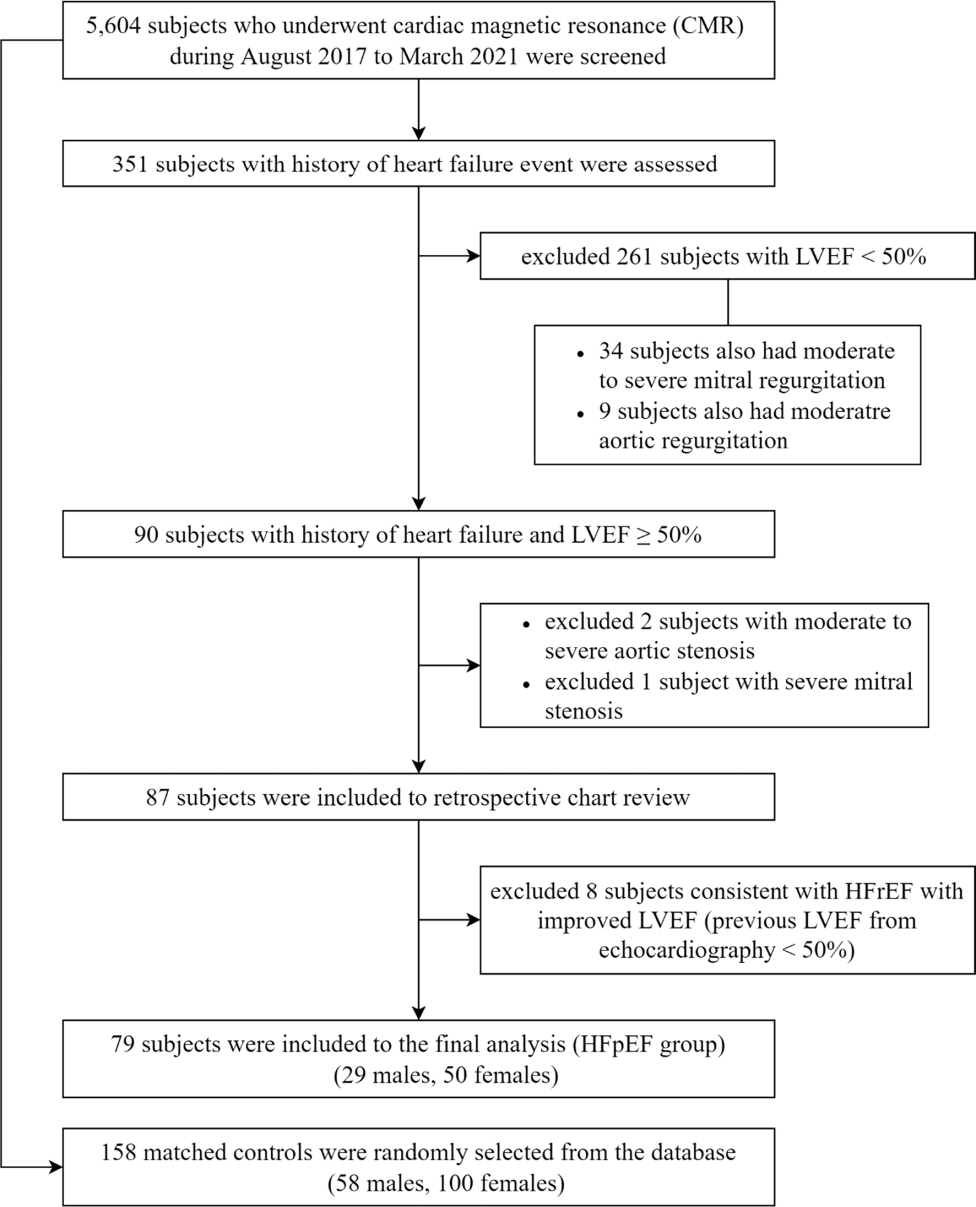
Flow diagram of the study protocol

Patients that underwent CMR during August 2017 to March 2021 and that satisfied all of the following criteria were consecutively included: 1) age ≥ 18 years, 2) history of at least 1 prior HF event, 3) LVEF ≥ 50%, and 4) was referred for adenosine stress or viability protocol CMR. Prior HF event was defined as new or worsening symptoms of HF with two types objective evidence from physical examination or one type of evidence from physical examination with one positive laboratory criterion, and receiving initiation or intensification of treatment specifically for HF [13]. The electronic database was use to filter out patients with exclusion criteria. The exclusion criteria included LVEF by CMR < 50% and moderate to severe left-sided valvular heart diseases including aortic or mitral stenosis and regurgitation. The electronic medical records of all included subjects were reviewed. Control subjects with LVEF ≥ 50% without a history of HF together with a negative ischemia result and absence of myocardial scar were selected from the same period and matched at a ratio of 2:1 (Fig. 1) using propensity scoring based on age, gender, and comorbidities.

### Image acquisition

All images were acquired using a 3-T magnetic resonance scanner (Ingenia; Philips Medical System, Best, the Netherlands) with a 32-channel dStream coil. The standard protocol included 1) black blood axial images; 2) SSFP cine images in a short axis stack with complete LV coverage, and 2-, 3-, and 4-chamber long axis views; 3) adenosine-stress perfusion scan; and, 4) LGE images. SSFP sequences were acquired during expiratory breath-holds with retrospective ECG gating. All cine images were acquired in 25 phases per cardiac cycle with 8 mm slice thickness without gap. The average parameters were, as follows: field of view 270 × 320 mm^2^, echo time 1.4 ms, repetition time 2.9 ms, flip angle 45°, and acquired voxel size 1.5 × 1.4 × 8 mm^3^. The temporal resolution was 34 ± 6 ms.

### Image post-processing

Image post-processing and analysis was performed using CVI42 software version 5.12 (Circle Cardiovascular Imaging, Calgary, Alberta, Canada). The primary observer who performed the feature tracking analysis was blinded to the subject group and patient comorbidities. Basic CMR parameters were measured using software-based semi-automated function analysis. Parameters were indexed for body surface area when appropriate.

### LV feature tracking analysis

**Fig. 2:**
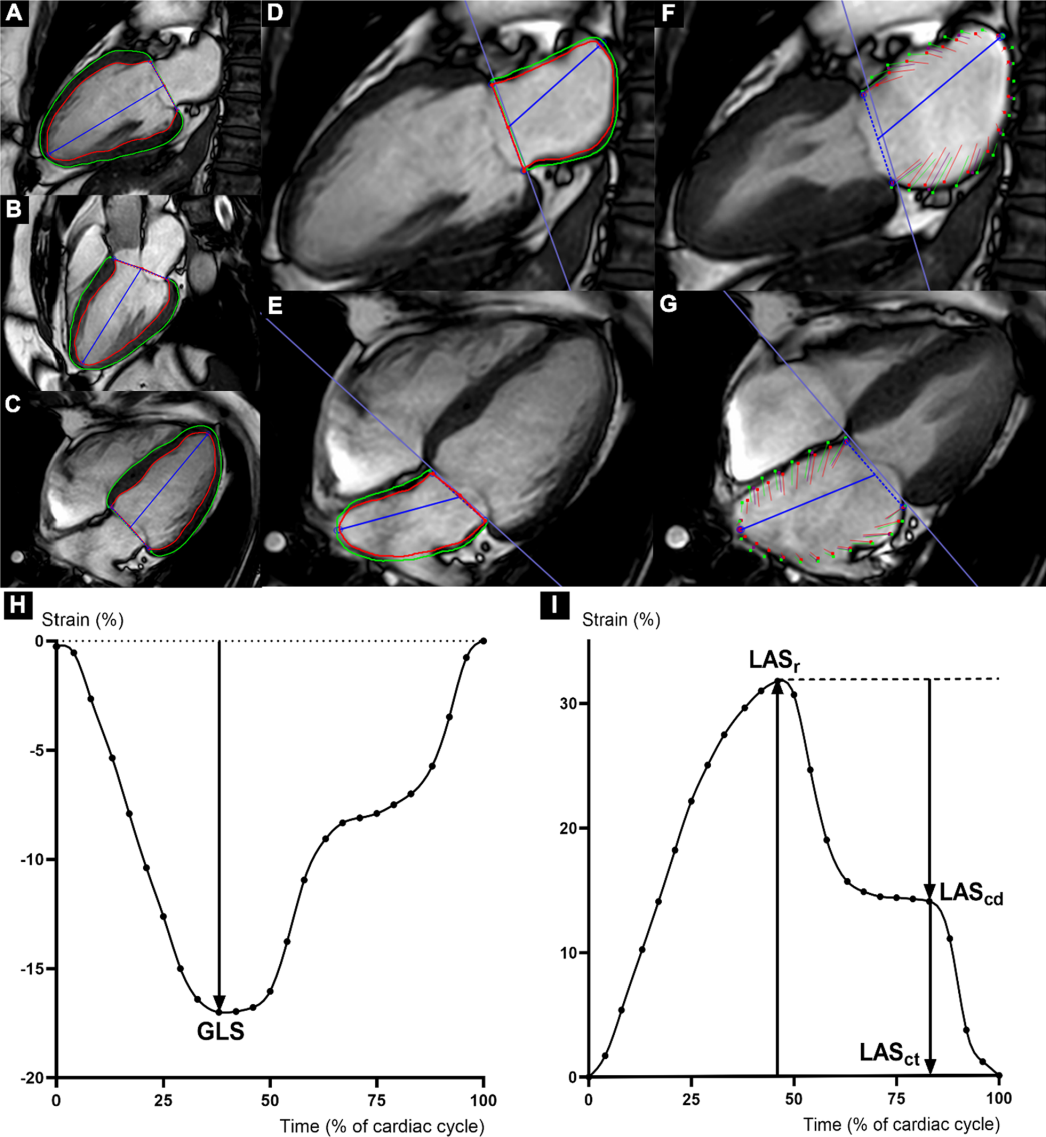
Left ventricular and atrial contours demonstrated in long axis images from cardiac magnetic resonance feature tracking (CMR-FT). Left ventricular (LV) endocardial (red line) and epicardial (green line) borders were semi-automatically drawn in 2-, 3-, and 4-chamber cine images (**A**–**C**) at the end-diastolic phase, while left atrial (LA) strain contours were manually drawn in 2- and 4-chamber cine images (**D**, **E**) at the end-diastole phase as displayed. In-plane motion of each voxel point within the region of interest was subsequently tracked by the software. Examples of point tracking at peak systolic phase are displayed on both the epicardial (green dots and lines) and endocardial (red dots and lines) borders of LV (Additional file 2–4) and LA (**F**, **G**; Additional file 5–6). The dots represent the current position of tracked voxel points, and the lines represent deformation from the baseline images. Longitudinal strain values were derived from the time-strain curves of LV global longitudinal strain (GLS) (**H**) and LA strain, including left atrial strain during reservoir phase (LAS_r_), left atrial strain during conduit phase (LAS_cd_), left atrial strain during contraction phase (LAS_ct_) (**I**)

The LV endocardial and epicardial borders were defined using software-based auto contour detection in 2-, 3-, and 4-chamber long axis cine images (Fig. 2 and Additional file 2–4), and in all slices of short axis cine images with a complete circumference of myocardium (Additional file 1: Figure S1) with carefully exclusion of papillary muscle and blood pool. Two-dimensional (2D) feature tracking was analyzed by the software with manual corrections being made, as needed. The peak value of global longitudinal strain (GLS) was derived from 3 long axis images (Fig. 2K), while the peak values of global circumferential strain (GCS) and global radial strain (GRS) were derived from short axis images (Additional file 1: Fig. 1). The definitions of these LV strain parameters were previously described in greater detail [7, 14].

To determine the difference in LA function in HFpEF using LV strain, the HFpEF group was subdivided into the lower strain and the higher strain groups for GLS, GCS, and GRS using the median of each LV strain value as the cut-off. Strain values of less negative or less positive than the median, depending on each strain types, were categorized into the lower strain group for each analysis i.e., lower GLS means less negative GLS value; lower GCS means less negative GCS value; and lower GRS means less positive GRS value. Lower strain indicates abnormality in each strain type.

### LA volume and function analysis

The LA endocardial border was manually traced with exclusion of pulmonary veins and atrial appendage [15, 16] on 2- and 4-chamber cine images during the ventricular end-diastolic and end-systolic phases to measure minimum LA volume (LAV_min_) and maximum LA volume (LAV_max_), respectively. LA volume was derived by software-based calculation using the bi-plane area-length method [16–18]. LAV_min_ and LAV_max_ were also indexed for BSA. LAEF was used to represent the total emptying function of the LA, whereas LA expansion index was used to represent reservoir function. Phasic LA emptying fraction (i.e., passive LAEF and active LAEF) and conduit volume were also measured. The formula for calculating these parameters were previously described [1, 4, 19].

### LA feature tracking analysis

The endocardial and epicardial borders were manually traced on 2- and 4-chamber long axis images with extrapolation across pulmonary veins and the atrial appendage orifice [20–22] (Fig. 2 and Additional file 5–6). Ventricular end-diastole was defined as the zero-strain reference according to EACVI/ASE/Industry Task Force [20]. Automatic 2D feature tracking was then applied (Additional file 5–6) and the initial contours were carefully readjusted to attain a sufficient border for tracking. Peak strain value and strain value before the atrial contraction phase were identified from the derived longitudinal strain–time curve (Fig. 2L). The different types of longitudinal LA strain were then analyzed to obtain each phasic LA strain corresponding to its function [1] [i.e., strain during reservoir phase (LAS_r_), strain during conduit phase (LAS_cd_), and strain during contraction phase (LAS_ct_)] using the following nomenclature and definitions published by the task force [20]:

- LAS_r_, the difference between peak strain value and reference (positive value)
- LAS_cd_, the difference between pre-atrial contraction strain value and peak strain value (negative value)
- LAS_ct_, the difference between pre-atrial contraction strain value and reference (negative value) (was not measured in patients with persistent atrial fibrillation)

### Statistical analysis

All statistical analyses were performed using SPSS 21.0 software (SPSS, Inc., Chicago, IL, USA). Baseline characteristics and basic CMR parameters were compared between the HFpEF group and controls using independent *t*-test. LA volume and mechanical parameters were tested for differences among the lower strain group, the higher strain group, and controls using one-way analysis of variance (ANOVA) and post hoc pairwise comparisons with Bonferroni correction. For each LA parameter, multiple linear regression was performed to adjust for age, gender, LVEF, LV mass index (LVMI), LGE status (presence or absence of scar), and heart rate. Reported cut-off for abnormal GLS (value of less negative than − 16%) [23], abnormal LA volume index (LAVi) (> 34 ml/m^2^), abnormal LAEF (total LAEF < 50%), and abnormal LAS_r_ (value of less positive than 23%) [24] were also assessed for sensitivity and specificity to determine HFpEF and association with the New York Heart Association (NYHA) functional classification adjusted for age, gender, and atrial fibrillation (AF) using logistic regression analysis.

Other categorical variables were compared using chi-square test or Fischer’s exact test, as appropriate. Correlations are described using Pearson’s correlation coefficient (*r*). Categorical variables are expressed as the number and percentage of subjects, and continuous variables as mean ± standard deviation (SD). Statistical significance was defined as a 2-tailed *p-*value of less than 0.05.

## Results

A total number of 79 HFpEF patients and 158 matched controls were included in the final analysis (Fig. 1). Most cine images were of sufficient quality for feature tracking analysis except 1 subject in the HFpEF group due to the presence of artifacts. The mean age of total subjects was 70.9 ± 10.5 years, and most subjects were female (63.3%). The average LVEF was 70.5 ± 7.0% (range: 52.6–86.5%). Baseline characteristics of the HFpEF group and the control group are given in Table 1. The HFpEF group had a higher prevalence of AF, and 7 of those patients still had AF rhythm during CMR scan. Mitral regurgitation was also more common in HFpEF group. Most of HFpEF patients were in NYHA class II. Diuretic use was significantly more common in the HFpEF group than in the control group, while other cardiovascular medications were not significantly different between groups. Diagnoses of patients in the HFpEF group that had been made by CMR were coronary artery disease (CAD) (26.6%), hypertrophic cardiomyopathy (13.9%), LV noncompaction (3.5%), and amyloidosis (2.5%). Ischemic-pattern scar as assessed by late-gadolinium enhancement (LGE) visualization was presented in 16.5%. However, the majority of HFpEF patients in this study (53.2%) did not fulfill any of the specific criteria for myocardial disease with the absence of myocardial scar.

**Table 1:** Baseline characteristics compared between HFpEF and controls

Characteristics	HFpEF (n = 79)	Controls (n = 158)	*p-*value
Age (years)	71.6 ± 11.5	70.6 ± 10.0	0.491
Male gender	29 (36.7%)	58 (36.7%)	1.000
Body mass index (kg/m^2^)	26.8 ± 5.6	26.6 ± 4.6	0.767
NYHA-II	68 (86.1%)	–	–
NYHA-III	11 (13.9%)	–	–
Hyperlipidemia	51 (64.6%)	116 (73.4%)	0.159
Diabetes	44 (55.7%)	85 (53.8%)	0.782
Hypertension	61 (77.2%)	114 (72.2%)	0.403
History of myocardial infarction	5 (6.3%)	0 (0.0%)	0.164
History of revascularization	7 (8.9%)	0 (0%)	0.333
History of atrial fibrillation	19 (24.1%)	2 (1.3%)	** < *0.001***
Mitral regurgitation	28 (35.4%)	21 (13.3%)	** < *0.001***
Medication use^†^			
Aspirin	28 (43.1%)	63 (47.4%)	0.569
Statin	43 (66.2%)	95 (71.4%)	0.448
β-blockers	38 (58.5%)	77 (57.9%)	0.939
ACEi	9 (13.8%)	16 (12.0%)	0.718
ARB	18 (27.7%)	42 (31.6%)	0.576
Diuretics	27 (41.5%)	31 (23.3%)	***0.008***

Data presented as mean ± standard deviation or number and percentage

*ACEi* angiotensin-converting enzyme inhibitors; *ARB* angiotensin receptor blockers; *HFpEF* heart failure with preserved ejection fraction; *NYHA* New York Heart Association functional class

A *p-*value < 0.05 indicates statistical significance (bold and italic)

^†^Medication data were available for 83.5% (n = 198) of total subjects

All types of LV strain (GLS, GCS, and GRS), the maximum and minimum LA volume index (LAVi_max_, LAVi_min_), and all LA mechanical parameters (total LAEF, passive LAEF, active LAEF, LA expansion index, LAS_r_, LAS_cd_, and LAS_ct_) were significantly different between HFpEF and controls (Table 2). GLS had a moderate negative correlation with total LAEF, LA expansion index, and LAS_r_ (Pearson’s correlation coefficient (*r*) of − 0.406, − 0.406, and − 0.500, respectively). Of the 3 types of LV strain, GLS was the most closely correlated with evaluated parameters. All 3 LV strain parameters had a better correlation with LAS_r_ than with LAS_ct_, LAS_cd_, and total LAEF (Additional file 1: Table S1).

**Table 2:** CMR parameters compared between HFpEF and controls

Parameters	HFpEF (n = 79)	Controls (n = 158)	*p-*value
Basic parameters			
LVEDV index (ml/m^2^)	66.6 ± 14.9	64.8 ± 12.1	0.315
LVESV index (ml/m^2^)	21.3 ± 8.2	18.9 ± 6.3	***0.013***
LVEF (%)	68.6 ± 7.6	71.5 ± 6.3	***0.002***
LVMI (g/m^2^)	64.0 ± 19.7	53.2 ± 10.8	** < *0.001***
RVEDV index (ml/m^2^)	64.1 ± 16.8	65.6 ± 13.9	0.461
RVEF (%)	57.5 ± 10.0	56.6 ± 8.5	0.467
LV strain parameters			
GLS (%)	− 13.3 ± 3.4	− 15.4 ± 2.2	** < *0.001***
GCS (%)	− 16.4 ± 3.6	− 18.9 ± 2.8	** < *0.001***
GRS (%)	28.1 ± 8.8	34.3 ± 8.1	** < *0.001***
LA volume and mechanical parameters			
LAVi_max_ (ml/m^2^)	45.1 ± 21.1	35.9 ± 10.3	** < *0.001***
LAVi_min_ (ml/m^2^)	27.3 ± 20.9	16.7 ± 7.2	** < *0.001***
LAEF, total (%)	43.5 ± 16.8	54.2 ± 12.2	** < *0.001***
LAEF, passive (%)	20.8 ± 9.6	26.2 ± 9.5	** < *0.001***
LAEF, active (%)	25.5 ± 11.1	28.6 ± 9.1	***0.043***
LA expansion index (%)	92.8 ± 57.2	132.0 ± 54.4	** < *0.001***
Conduit volume index (ml/m^2^)	27.5 ± 8.3	26.1 ± 9.3	0.236
LAS_r_ (%)	19.5 ± 9.7	26.7 ± 8.8	** < *0.001***
LAS_cd_ (%)	− 9.7 ± 5.9	− 14.8 ± 7.4	** < *0.001***
LAS_ct_ (%)	− 10.7 ± 5.2	− 12.4 ± 4.3	***0.017***
Abnormal GLS (≥ − 16%)	59 (75.6%)	87 (56.1%)	***0.004***
Abnormal LAVI (> 34 ml/m2)	54 (68.4%)	84 (53.5%)	***0.029***
Abnormal LASr (< 23%)	49 (62.0%)	60 (38.5%)	***0.001***
Abnormal LAEF (< 50%)	47 (59.5%)	43 (27.4%)	** < *0.001***
Heart rate (beats per minute)	73.0 ± 13.5	71.0 ± 12.6	0.237

Data presented as mean ± standard deviation or number and percentage

*CMR* indicates cardiac magnetic resonance; *HFpEF* heart failure with preserved ejection fraction; *GCS* global circumferential strain; *GLS* global longitudinal strain; *GRS* global radial strain; *LAVi*_*max*_ maximal left atrial volume index; *LAVi*_*min*_ minimal left atrial volume index; *LAEF* left atrial emptying fraction; *LAS*_*cd*_ left atrial strain during conduit phase; *LAS*_*ct*_ left atrial strain during contraction phase; *LAS*_*r*_ left atrial strain during reservoir phase; *LVEDV* left ventricular end-diastolic volume; *LVEF* left ventricular ejection fraction; *LVESV* left ventricular end-systolic volume; *LVMI* LV mass index; *RVEDV* right ventricular end-diastolic volume; *RVEF* right ventricular ejection fraction

A *p-*value < 0.05 indicates statistical significance (bold and italic)

### LA volume and mechanical parameters compared between HFpEF subgroups stratified by type of LV strain and controls

**Fig. 3:**
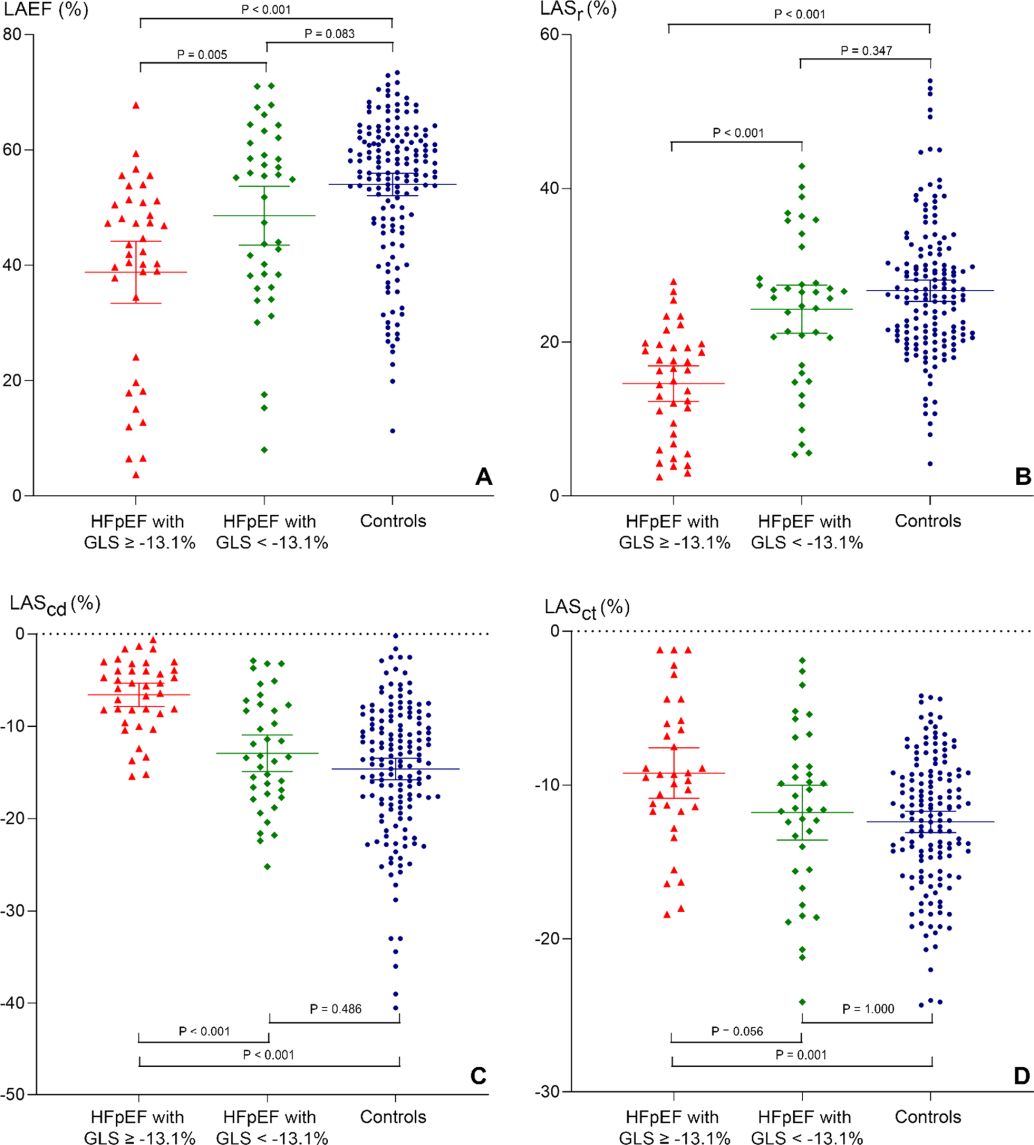
Comparison of left atrial (LA) mechanical parameters. LA emptying fraction (LAEF) (**A**), left atrial strain during reservoir phase (LAS_r_) (**B**), left atrial strain during conduit phase (LAS_cd_) (**C**), and left atrial strain during contraction phase (LAS_ct_) (**D**) were compared between heart failure with preserved ejection fraction (HFpEF) with global longitudinal strain (GLS) ≥ median and controls, and between HFpEF with GLS < median and controls. The central horizontal line represents the mean of each group with 95% confidence interval. A *p-*value of less than 0.05 indicates a statistically significant difference between the means of each group

The median GLS, GCS, and GRS values in the HFpEF group were − 13.1%, − 16.6%, and 27.9%, respectively. Patients in the HFpEF group were stratified into 2 subgroups according to the median of each strain, as follows: 1) HFpEF with lower GLS (value ≥ -13.1%) and higher GLS (value <  − 13.1%), 2) HFpEF with lower GCS (value ≥ -16.6%) and higher GCS (value < 16.6%), and 3) HFpEF with lower GRS (value < 27.9%) and higher GRS (value ≥ 27.9%). Total LAEF, LAS_r_, and LAS_cd_ were all significantly impaired in HFpEF patients with significantly more impairment in the lower GLS group than in the higher GLS group, and than in the control group (Fig. 3). Mean LAS_ct_ was only significantly different between HFpEF with higher GLS and controls, but not between the lower and higher GLS subgroups. All LA parameters remained significantly different after adjusting for age, gender, LVEF, LVMI, LGE status, and heart rate (Additional file 1: Table S2).

When HFpEF patients were stratified by GCS and GRS, LA volume and mechanical parameters except for total LAEF and LA expansion index of the lower strain group were significantly different from other groups. All LA parameters remained significantly different after adjusting for age, gender, LVEF, LVMI, LGE status, and heart rate (Additional file 1: Table S3 and S4).

Sensitivity analyses were performed by 1) excluding the patients with AF, and 2) excluding the patients with positive LGE. The results of that analysis showed that HFpEF with a GLS value ≥  − 13.1% still had significantly impaired LAS_r_ and LAS_cd_ compared to both HFpEF with a GLS value <  − 13.1% and controls (LAS_r_: 17.7 ± 5.3% *vs.* 26.9 ± 7.8% [*p* < 0.001] and 26.9 ± 8.7% [*p* < 0.001], respectively; LAS_cd_: − 7.4 ± 3.9% *vs.* − 14.4 ± 5.3% [*p* < 0.001] and − 14.8 ± 7.3% [*p* < 0.001], respectively), and after excluding patients with positive LGE (LAS_r_: 15.2 ± 7.5% vs. 24.3 ± 10.2% [*p* = 0.002] and 26.7 ± 8.8% [*p* < 0.001], respectively; LAS_cd_: − 8.0 ± 4.5% vs. − 13.3 ± 6.2% [*p* = 0.029] and − 14.6 ± 7.4% [*p* < 0.001], respectively)—all independent of the aforementioned potential confounders.

### Prevalence of abnormal LA mechanical parameters and their association with clinical symptoms

Abnormal LAVi (> 34 ml/m2), abnormal LAEF (< 50%), and abnormal LAS_r_ (< 23%) were more prevalent in HFpEF group than control group (Table 2). Abnormal LAEF and abnormal LAS_r_ were also more common in patients with arterial hypertension or CAD (Additional file 1: Table S6 and Table S7). Abnormal LAVi, LAEF, and LAS_r_ were associated with NYHA class ≥ II when analyzing the whole cohort and a subgroup of patients with presence of at least one cardiovascular risk factor or established CAD (Table 3). When adjusting for age, gender, and AF, only abnormal LAEF and LAS_r_ were independently associated with NYHA class ≥ II. Comparing with abnormal LAVi, abnormal GLS (≥ -16%) had a higher sensitivity (75.6% versus 43.9%) while abnormal LAEF and abnormal LAS_r_ had a higher specificity (72.6% and 62.5% respectively, versus 46.5%) with a slightly lower sensitivity (59.5% and 62.0% respectively, versus 68.4%) to determine HFpEF. When combining abnormal LAVi with abnormal LAEF or abnormal LAS_r_, the specificity was improved than using abnormal LAVi alone (75.6% versus 46.5%) (Additional file 1: Table S8).

**Table 3:** Odds ratio of abnormal LAVi, LAEF and LAS_r_ to determine patients with NYHA class ≥ II

Variables	Odds ratio (95% CI)	*p*-value	Odds ratio (95% CI) ^†^	*p*-value
Analysis of the whole cohort				
LAVi > 34 ml/m^2^	1.877 (1.063–3.314)	**0.030**	1.297 (0.708–2.375)	0.400
LAEF < 50%	3.894 (2.202–6.885)	** < 0.001**	3.382 (1.791–6.389)	** < *0.001***
LAS_r_ < 23%	2.613 (1.497–4.562)	**0.001**	2.064 (1.118–2.110)	***0.021***
Analysis of the patients with at least one CV risk factor or CAD				
LAVi > 34 ml/m^2^	2.018 (1.121–3.634)	**0.019**	1.416 (0.757–2.646)	0.276
LAEF < 50%	3.953 (2.197–7.114)	** < 0.001**	3.340 (1.751–6.370)	** < *0.001***
LAS_r_ < 23%	2.410 (1.319–4.274)	**0.003**	1.887 (1.006–3.537)	***0.048***

A *p*-value < 0.05 indicates statistical significance (bold and italic)

^†^Odds ratio were adjusted for age, gender, and AF

*LAVi* left atrial volume index; *LAEF* LA emptying fraction; *LAS*_*r*_, LA strain during reservoir phase; *NYHA* New York Heart Association functional class

### Patient outcomes

Of those 79 HFpEF patients, 64 patients (81.0%) had a follow-up data. During a median follow-up of 25.3 months from the date of CMR study (range 4.6–41.2 months), 7 patients (10.9%) had a recurrent heart failure event that required hospitalization. Five of 7 patients were from HFpEF with lower GLS (median time-to-event 6.3 months [range 1.6–25.4 months]), and 2 patients were from HFpEF with higher GLS (time-to-event 6.0 and 17.2 months). Another patient from HFpEF with lower GLS also had a cardiovascular death from fatal arrhythmia (time-to-event 12.9 months). Due to the small number of events, survival analysis was not conducted.

### Intraobserver and interobserver agreement

Two samples, each consisting of 25 subjects (10% of the total number of subjects), were randomly selected with a 1:1 ratio between the HFpEF group and the control group. Evaluation of the different types of LV strain (GLS, GCS, and GRS) and the different types of LA strain (LAS_r_, LAS_cd_, and LAS_ct_) was performed separately by the primary observer and another observer (who was blinded to the objective of this study) to evaluate reproducibility. Overall, GLS had the best intra- and interobserver agreement (bias: 0.36 ± 1.30 and 0.86 ± 1.31, respectively). Bland–Altman plots illustrating intra- and interobserver variability with limits of agreement estimation for each strain parameter were generated (Additional file 1: Figure S2 and S3).

## Discussion

The present study demonstrates the differences in LAVi_max_, LAVi_min_, total LAEF, LA expansion index, and each type of phasic LA strain (LAS_r_, LAS_cd_, and LAS_ct_) among HFpEF with lower LV strain, HFpEF with higher LV strain, and controls. We found that HFpEF with lower GLS had the highest LAVi_max_ and LAVi_min_ (surrogates for chronic LA remodeling), the lowest LA expansion index and LAS_r_ (reflecting abnormality in reservoir function), and the worst LAEF and LAS_cd_, (reflecting abnormality in total and passive emptying function, respectively). Interestingly, most of the LA volume and mechanical parameters were not significantly different between those with higher strain HFpEF and matched controls, which may indicate similar LA mechanics between higher strain HFpEF group and those without HF.

Abnormal LAEF (< 50%) and abnormal LAS_r_ (< 23%) were more prevalent in HFpEF and in subjects with arterial hypertension and CAD. Abnormal LAEF and abnormal LAS_r_ were independently associated with worse functional capacity (NYHA class ≥ II) in the matched cohort and in a subgroup with presence of at least one cardiovascular risk factor or established CAD. In contrast, the usual cut-off for abnormal LAVi (> 34 ml/m2) was not independent associated with NYHA class ≥ II after adjusting by age, gender, and AF in our population, and had a lower specificity compared to abnormal LAEF and abnormal LAS_r_ to determine HFpEF.

To the best of our knowledge, this is the first study to demonstrate different prevalence of abnormal LA mechanics LA mechanics in HFpEF with lower range versus higher range of LV strain assessed by CMR-FT technique. However, the correlation between LA strain and LV strain was previously reported in echocardiographic-based studies of LA strain. There is also a previous study that demonstrated a strong correlation between CMR and echocardiographic-based strain assessment in cardiomyopathy patients [25]. Our findings support evidence of LA strain impairment in HFpEF compared to controls, especially for LAS_r_ [17, 19, 26, 27], and LAS_cd_ [17, 26], and also provides additional insights in evaluation of LA mechanical impairment in HFpEF by CMR. This results are consistent with a previous study that showed a moderate correlation of LAEF and LAS_r_ with maximal oxygen uptake in HFpEF but not for the LA volume [26]. The moderate negative correlation between GLS and LAS_r_ in this study is also similar to another larger study which also found that LV function has an influence on the association between impaired LA function and higher risk of HF hospitalization [28]. These findings support the interrelated nature of the mechanical processes of both chambers. However, disproportionate LA malfunction from intrinsic LA abnormalities in some of HFpEF patients may be another factor that explains why the correlation of LAS_r_ and GLS is only moderate [29].

In contrast, a different study that used CMR-FT to compare LA function and strain found no different LA strain between HFpEF and controls [22]. These differences in results may be explained by the different definitions of zero-strain reference in the LA strain curve. In our study, the ventricular end-diastolic phase was the zero-strain reference, whereas another study used the onset of LA contraction as the zero-strain reference. According to EACVI/ASE/Industry Task Force recommendation, the ventricular end-diastole reference is currently recommended [20].

Compared with those from another study [17], our control subjects also had more negative LAS_ct_ values. The possible explanation this difference between studies is that the mean age of our controls was substantially higher than the mean age of controls in that study (70.6 *vs.* 40.6 years, respectively) [17]. A previous study in healthy adults found that the LA contraction-to-reservoir ratio increased significantly with age [30]. Another CMR-based study also found that active LA emptying fraction, which also reflects increased active emptying function, was increased with age in healthy volunteers [31]. Another possible explanation is the intervendor inconsistency.

### Pathophysiology of deranged LA mechanics in HFpEF

**Fig. 4:**
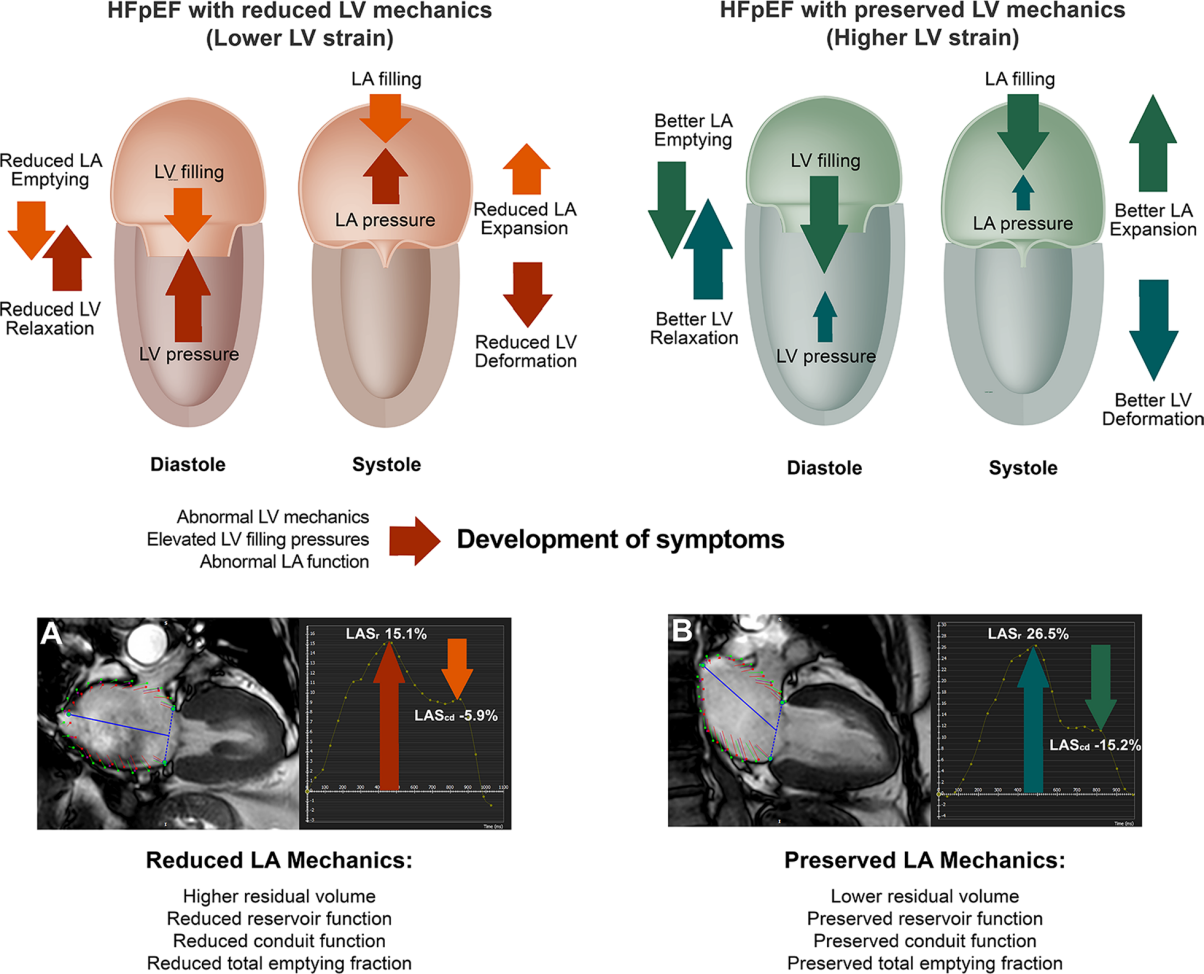
Left ventricular mechanics and effects on left atrial mechanics. Summary of mechanism that explain the relationship between left ventricular and left atrial mechanics in heart failure with preserved ejection fraction and its role in the development of symptoms; Examples of LA strain–time curve from of HFpEF with lower LV strain (**A**) and higher LV strain (**B**). *HFpEF* heart failure with preserved ejection fraction; *LA* left atrial; *LV* left ventricular

LA mechanical function consists of 3 phases, including reservoir, conduit (passive emptying), and contractile (active-emptying) function [1] which is also influenced by the contraction and compliance of the LV [1, 32, 33]. In HFpEF, abnormal relaxation of the LV increases the downstream pressure (the LA afterload), which eventually worsens the emptying function and LA compliance (Fig. 4). Abnormal LA function, in turn, is associated with elevated LV end-diastolic pressure [15] and LV diastolic dysfunction [10–12, 27]. Deterioration of atrial function leads to maladaptation of LA remodeling, LA enlargement, and subsequently results in pulmonary venous congestion [5], as well as more reduced exercise capacity [5, 26]. LA remodeling also generates substrates for AF, which is one of the important comorbidities of HFpEF [32], and drastically adversely affects LA compliance and mechanics [34]. After excluding patients with AF, the results also emphasize the significant impairment of LA mechanics even in patients who have not yet developed AF.

### Clinical implication and future direction

The results of this study provide evidence supporting the feasibility and benefit of a dedicated assessment of LA function and strain in patients with HFpEF [35]. Our study may support the usage of LAEF and LAS_r_ the concept of HFpEF staging by integration of LV and LA mechanics [6], and consistent with the knowledge of previously described HFpEF phenogroup 2 of TOPCAT (Treatment of Preserved Cardiac Function Heart Failure with an Aldosterone Antagonist) trial, of which pathophysiology is characterized by older age, marked LA enlargement, and high burden of diastolic dysfunction [36]. Moreover, LA strain assessment also predicts AF progression in HFpEF [34] and correlates with increased myocardial extracellular volume [37] which indicates accumulation of extracellular matrix in HFpEF [38]. Treatments that target cardiac fibrosis [39] and LA reverse remodeling [40] may have benefit in such population. Even though evidence supports the prognostic utility of CMR-FT-derived LV strain [41] and LA strain in HFpEF [22, 42], benefits of HFpEF evaluation according to integrated data of LV and LA mechanics (i.e. impaired LV strain with or without impaired LA function) are still unknown and should be investigated in future study. LAEF and LAS_r_ may have an additional benefit to increase diagnosis performance of LAVi to determine HFpEF in patients undergo CMR, especially those with NYHA class ≥ II.

### Study limitations

The present study has some limitations that need to be acknowledged. First, due to our study’s retrospective cross-sectional design, the diagnosis of HFpEF in this study was based solely on the decisions arrived at by primary physicians, and may not be consistent with a recently reported novel approach to diagnosing HFpEF [43]. Second, the evaluation for severity of valvular heart dysfunction in our center was mostly based on a qualitative assessment and may be less accurate compared to a dedicated quantitative assessment [44]. Third, all study subjects were selected from patients who had indications for a CMR scan. Thus, the results of all types of LV and LA strain in the control group may not be the same as healthy subjects. Fourth, our results are based on surrogates of LA physiology rather than on more accurate invasive physiologic study. Moreover, this study focused on global evaluation of LV strain; more advance diastolic features such as LV torsion or twist were thus omitted. Fifth and last, our study did not include an analysis of follow-up data to identify relationships and effects over time. As such, a larger prospective study is needed to evaluate the prognostic utility of CMR-FT in this specific HFpEF subgroup.

## Conclusions

Patients with HFpEF were found to have impaired LV and LA mechanics. Abnormal LA mechanics was more prevalent in HFpEF patients with lower LV strain and significantly associated with the symptomatic status of the patients.

## Supplementary Information

**Additional file 1**: Supplementary Data.

**Additional file 2**: Video representing point tracking of LV in 2-chamber view.

**Additional file 3**: Video representing point tracking of LV in 3-chamber view.

**Additional file 4**: Video representing point tracking of LV in 4-chamber view.

**Additional file 5**: Video representing point tracking of LA in 2-chamber view.

**Additional file 6**: Video representing point tracking of LA in 4-chamber view.

## Data Availability

The datasets used and/or analyzed during the current study are available from the corresponding author on reasonable request.

## Abbreviations

ANOVA: Analysis of variance
BSA: Body surface area
CMR: Cardiac magnetic resonance
CMR-FT: Cardiac magnetic resonance feature tracking
GCS: Global circumferential strain
GLS: Global longitudinal strain
GRS: Global radial strain
HF: Heart failure
HFpEF: Heart failure with preserved ejection fraction
LA: Left atrium, left atrial
LAEF: Left atrial emptying fraction
LAS_cd_: Left atrial strain during conduit phase
LAS_ct_: Left atrial strain during contraction phase
LAS_r_: Left atrial strain during reservoir phase
LAVi: Left atrial volume index
LAV_max_: Maximum left atrial volume
LAV_min_: Minimum left atrial volume
LGE: Late gadolinium enhancement
LV: Left ventricle, left ventricular
LVEDP: Left ventricular end-diastolic pressure
LVEDV: Left ventricular end-diastolic volume
LVEF: Left ventricular ejection fraction
LVESV: Left ventricular end-systolic volume
LVMI: Left ventricular mass index
RVEDV: Right ventricular end-diastolic volume
RVEF: Right ventricular ejection fraction
SSFP: Steady-state free precession
