# Supplementary material for: Impact of different degrees of left ventricular strain on left atrial mechanics in heart failure with preserved ejection fraction
Source: BMC Cardiovasc Disord. 2022 Apr 9;22:160. doi: 10.1186/s12872-022-02608-7 (PMC8994220; doi:10.1186/s12872-022-02608-7)
Supplement: Supplementary file 1 — Additional file 1: Supplementary Data. [file 12872_2022_2608_MOESM1_ESM.docx]

# Supplementary Data

**Table S1.** Correlation between LA function parameters and different types of LV strain

| **Parameters** | **GLS** | **GCS** | **GRS** |
| --- | --- | --- | --- |
| LAVi_max_ (ml/ m^2^) | 0.138 | 0.243 | -0.247 |
| LAVi_min_ (ml/ m^2^) | 0.312 | 0.340 | -0.336 |
| LAEF, total (%) | -0.406 | -0.362 | -0.341 |
| LA expansion index (%) | -0.406 | -0.362 | -0.341 |
| LAS_r_ (%) | -0.500 | -0.466 | 0.440 |
| LAS_cd_ (%) | 0.507 | 0.428 | -0.393 |
| LAS_ct_ (%) | 0.181 | 0.236 | -0.236 |

Values are Pearson’s correlation coefficients (*r*) (all presented coefficients have a *p-*value <0.05)

GLS indicates global longitudinal strain; GCS, global circumferential strain; GRS, global radial strain; LA, left atrial; LAVi_max_, maximal left atrial volume index; LAVi_min_, minimal LA volume index; LAEF, LA emptying fraction; LAS_cd_, LA strain during conduit phase, LAS_ct_, LA strain during contraction phase; LAS_r,_ LA strain during reservoir phase; LV, left ventricular.

**Table S2.** LA function parameters compared among HFpEF patients with GLS ≥ median, HFpEF patients with GLS < median, and controls

| **Variables** | **HFpEF with GLS ≥ -13.1% (n=39)** | **HFpEF with GLS < -13.1%**  **(n=39)** | **Controls**  **(n=158)** | **ANOVA**  ***p*-value** | **Adjusted**  ***p*-value^†^** |
| --- | --- | --- | --- | --- | --- |
| LAVI_max_ (ml/m^2^) | 49.7±25.4 | 40.9±14.9 | 35.9±10.3 | ***<0.001 ^a, b^*** | ***0.017*** |
| LAVI_min_ (ml/m^2^) | 32.7±24.7 | 22.0±15.1 | 16.7±7.7 | ***<0.001 ^a, b^*** | ***0.001*** |
| LAEF total (%) | 38.8±16.6 | 48.6±15.7 | 54.2±12.2 | ***<0.001 ^a, b^*** | ***<0.001*** |
| LA expansion index (%) | 74.7±44.8 | 112.3±62.6 | 132.0±54.4 | ***<0.001 ^a, b^*** | ***<0.001*** |
| LAS_r_ (%) | 14.6±7.1 | 24.3±9.6 | 26.7±8.8 | ***<0.001 ^a, b^*** | ***<0.001*** |
| LAS_cd_ (%) | -6.6±3.9 | -12.9±6.0 | -14.7±7.4 | ***<0.001 ^a, b^*** | ***0.001*** |
| LAS_ct_ (%) | -9.2±4.7 | -11.8±5.3 | -12.4±4.3 | ***0.001^b^*** | ***0.002*** |

Data presented as mean ± standard deviation

A *p*-value<0.05 indicates statistical significance

^†^ *P*-values were adjusted for age, gender, LVEF, LVMI, LGE status, and heart rate

^a^ Post hoc significant difference between HFpEF with GLS ≥ median and HFpEF with GLS < median

^b^ Post hoc significant difference between HFpEF with GLS ≥ median and controls

**Abbreviations:** ANOVA, analysis of variance; HFpEF, heart failure with preserved ejection fraction; GLS, global longitudinal strain; LA, left atrial; LAVI_max_, maximal left atrial volume index; LAVI_min_, minimal LA volume index; LAEF, LA emptying fraction; LAS_r,_ LA strain during reservoir phase; LAS_cd_, LA strain during conduit phase, LAS_ct_; LA strain during contraction phase

**Table S3.** LA function parameters compared among HFpEF patients with GCS ≥ median, HFpEF patients with GCS < median, and controls

| **Parameters** | **HFpEF with**  **GCS ≥ -16.6%**  **(n=40)** | **HFpEF with**  **GCS < -16.6%**  **(n=39)** | **Controls**  **(n=158)** | **ANOVA**  ***p-*value** | **Adjusted**  ***p-*value^†^** |
| --- | --- | --- | --- | --- | --- |
| LAVi_max_ (ml/m^2)^ | 50.1±24.9 | 40.0±14.9 | 35.9±10.3 | ***<0.001 ^a, b^*** | ***0.013*** |
| LAVi_min_ (ml/m^2)^ | 32.3±24.5 | 22.1±15.1 | 16.7±7.7 | ***<0.001 ^a, b^*** | ***0.001*** |
| LAEF total (%) | 40.2±16.0 | 46.9±17.1 | 54.2±16.2 | ***<0.001 ^b, c^*** | ***<0.001*** |
| LA expansion index (%) | 78.6±45.5 | 107.3±64.7 | 132.0±54.4 | ***<0.001 ^b, c^*** | ***0.001*** |
| LAS_r_ (%) | 15.5±8.0 | 23.6±9.6 | 26.7±8.8 | ***<0.001 ^a, b^*** | ***<0.001*** |
| LAS_cd_ (%) | -6.8±4.1 | -12.7±6.1 | -14.7±7.4 | ***<0.001 ^a, b^*** | ***0.002*** |
| LAS_ct_ (%) | -10.0±5.0 | -11.3±5.4 | -12.4±4.3 | ***0.002 ^b^*** | ***0.018*** |

Data presented as mean ± standard deviation

A *p-*value<0.05 indicates statistical significance

^†^ *P*-values were adjusted for age, gender, LVEF, LVMI, LGE status, and heart rate

^a^ Post hoc significant difference between HFpEF with GCS ≥ median and HFpEF with GCS < median

^b^ Post hoc significant difference between HFpEF with GCS ≥ median and controls

^c^ Post hoc significant difference between HFpEF with GCS < median and controls

**Abbreviations:** ANOVA, analysis of variance; HFpEF, heart failure with preserved ejection fraction; GLS, global longitudinal strain; LA, left atrial; LAVi_max_, maximal left atrial volume index; LAVi_min_, minimal LA volume index; LAEF, LA emptying fraction; LAS_r,_ LA strain during reservoir phase; LAS_cd_, LA strain during conduit phase, LAS_ct_; LA strain during contraction phase

**Table S4.** LA function parameters compared among HFpEF patients with GRS < median, HFpEF patients with GRS ≥ median, and controls

| **Parameters** | **HFpEF with**  **GRS < 27.9%**  **(n=39)** | **HFpEF with**  **GRS ≥ 27.9%**  **(n=40)** | **Controls**  **(n=158)** | **ANOVA**  ***p-*value** | **Adjusted**  ***p-*value^†^** |
| --- | --- | --- | --- | --- | --- |
| LAVi_max_, (ml/ m^2)^ | 51.8±24.2 | 38.5±15.1 | 35.9±10.3 | ***<0.001 ^a, b^*** | ***0.004*** |
| LAVi_min_, (ml/ m^2)^ | 33.6±24.4 | 21.1±14.8 | 16.7±7.7 | ***<0.001 ^a, b^*** | ***<0.001*** |
| LAEF total (%) | 40.0±16.5 | 46.9±16.5 | 54.2±12.2 | ***<0.001 ^b, c^*** | ***<0.001*** |
| LA expansion index (%) | 78.8±47.4 | 106.5±63.1 | 132.0±54.4 | ***<0.001 ^b, c^*** | ***0.001*** |
| LAS_r_ (%) | 15.8±8.3 | 23.2±9.6 | 26.7±8.8 | ***<0.001 ^a, b^*** | ***<0.001*** |
| LAS_cd_ (%) | -7.3±4.4 | -2.0±6.4 | -14.7±7.4 | ***<0.001 ^a, b^*** | ***0.005*** |
| LAS_ct_ (%) | -9.7±5.1 | -11.5±5.2 | -12.4±4.3 | ***0.010 ^b^*** | ***0.013*** |

Data presented as mean ± standard deviation

A *p-*value<0.05 indicates statistical significance

^†^ *P*-values were adjusted for age, gender, LVEF, LVMI, LGE status, and heart rate

^a^ Post hoc significant difference between HFpEF with GRS ≥ median and HFpEF with GRS < median

^b^ Post hoc significant difference between HFpEF with GRS < median and controls

^c^ Post hoc significant difference between HFpEF with GRS ≥ median and controls

ANOVA indicates analysis of variance; HFpEF, heart failure with preserved ejection fraction; GLS, global longitudinal strain; LA, left atrial; LAVi_max_, maximal left atrial volume index; LAVi_min_, minimal LA volume index; LAEF, LA emptying fraction; LAS_r,_ LA strain during reservoir phase; LAS_cd_, LA strain during conduit phase, LAS_ct_; LA strain during contraction phase

**Table S5.** LA function parameters compared among HFpEF patients with lower GLS (GLS value ≥ -16.0, HFpEF patients with lower GLS (GLS value < -16.0%), and controls

| **Variables** | **HFpEF with GLS ≥ -13.1% (n=39)** | **HFpEF with GLS < -13.1%**  **(n=39)** | **Controls**  **(n=158)** | **ANOVA**  ***p*-value** | **Adjusted**  ***p*-value^†^** |
| --- | --- | --- | --- | --- | --- |
| LAVI_max_ (ml/m^2^) | 46.6±22.4 | 41.1±16.5 | 35.9±10.3 | ***<0.001 ^b^*** | ***0.046*** |
| LAVI_min_ (ml/m^2^) | 29.4±21.9 | 21.0±17.1 | 16.7±7.7 | ***<0.001 ^b^*** | ***0.003*** |
| LAEF total (%) | 41.3±16.4 | 51.2±16.1 | 54.2±12.1 | ***<0.001 ^a, b^*** | ***<0.001*** |
| LA expansion index (%) | 83.5±50.6 | 124.5±66.7 | 132.0±54.4 | ***<0.001 ^a, b^*** | ***<0.001*** |
| LAS_r_ (%) | 17.7±9.4 | 24.8±9.1 | 26.7±8.8 | ***<0.001 ^a, b^*** | ***0.001*** |
| LAS_cd_ (%) | -8.4±5.3 | -13.7±6.2 | -14.7±7.4 | ***<0.001 ^a, b^*** | ***0.006*** |
| LAS_ct_ (%) | -10.4±5.1 | -11.0±5.4 | -12.4±4.3 | ***0.023 ^b^*** | ***0.032*** |

Data presented as mean ± standard deviation

A *p*-value<0.05 indicates statistical significance

^†^ *P*-values were adjusted for age, gender, LVEF, LVMI, LGE status, and heart rate

^a^ Post hoc significant difference between HFpEF with GLS ≥ median and HFpEF with GLS < median

^b^ Post hoc significant difference between HFpEF with GLS ≥ median and controls

**Abbreviations:** ANOVA, analysis of variance; HFpEF, heart failure with preserved ejection fraction; GLS, global longitudinal strain; LA, left atrial; LAVI_max_, maximal left atrial volume index; LAVI_min_, minimal LA volume index; LAEF, LA emptying fraction; LAS_r,_ LA strain during reservoir phase; LAS_cd_, LA strain during conduit phase, LAS_ct_; LA strain during contraction phase

**Table S6.** Prevalence of abnormal LAS_r_ in patients with cardiovascular risk factors or established CAD.

| **Parameters** | **Whole cohort (n=235)** | | **Patient with sinus rhythm (n=236)** | |
| --- | --- | --- | --- | --- |
|  | **Frequency (%)** | ***p-*value** | **Frequency (%)** | ***p-*value** |
| - Diabetes | 74 (57.4%) | <0.001 | 64 (55.2%) | <0.001 |
| - No diabetes | 35 (33.0%) |  | 27 (27.6%) |  |
| - Arterial hypertension | 96 (55.2%) | <0.001 | 81 (51.6%) | <0.001 |
| - No arterial hypertension | 13 (21.3%) |  | 10 (17.5%) |  |
| - Obesity (BMI ≥ 25 kg/m^2^) | 74 (51.4%) | 0.053 | 62 (47.0%) | 0.095 |
| - No obesity (BMI < 25 kg/m^2^) | 35 (38.5%) |  | 29 (35.4%) |  |
| - Established CAD | 17 (81.0%) | 0.001 | 11 (78.6%) | 0.009 |
| - No established CAD | 92 (43.0%) |  | 80 (40.0%) |  |

A *p-*value<0.05 indicates statistical significance

BMI indicates body mass index; CAD, coronary artery disease; LAS_r,_ LA strain during reservoir phase.

**Table S7.** Prevalence of abnormal LAEF in patients with cardiovascular risk factors or established CAD.

| **Parameters** | **Whole cohort (n=235)** | | **Patient with sinus rhythm (n=236)** | |
| --- | --- | --- | --- | --- |
|  | **Frequency (%)** | ***p-*value** | **Frequency (%)** | ***p-*value** |
| - Diabetes | 53 (41.1%) | 0.306 | 44 (37.9%) | 0.182 |
| - No diabetes | 37 (34.6%) |  | 29 (29.3%) |  |
| - Arterial hypertension | 77 (44.3%) | 0.001 | 63 (40.1%) | 0.002 |
| - No arterial hypertension | 13 (21.0%) |  | 10 (17.2%) |  |
| - Obesity (BMI ≥ 25 kg/m^2^) | 56 (38.9%) | 0.766 | 44 (33.3%) | 0.809 |
| - No obesity (BMI < 25 kg/m^2^) | 34 (37.0%) |  | 29 (34.9%) |  |
| - Established CAD | 16 (76.2%) | <0.001 | 11 (78.6%) | 0.001 |
| - No established CAD | 74 (34.4%) |  | 62 (30.8%) |  |

A *p-*value<0.05 indicates statistical significance

BMI indicates body mass index; CAD, coronary artery disease; LAEF_,_ left atrial emptying fraction.

**Table S8.** Sensitivity and specificity of abnormal LA parameters to determine HFpEF

| **Parameters** | **Sensitivity** | **Specificity** |
| --- | --- | --- |
| Abnormal GLS (≥-16%) | 0.756 | 0.439 |
| Abnormal LAVi (>34 ml/m2) | 0.684 | 0.465 |
| Abnormal LAS_r_ (<23%) | 0.620 | 0.615 |
| Abnormal LAEF (<50%) | 0.595 | 0.726 |
| Abnormal LAVi + Abnormal LAS_r_ or LAEF | 0.481 | 0.756 |
| Abnormal LAS_r_ or Abnormal LAEF | 0.722 | 0.591 |
| Abnormal LAS_r_ + Abnormal LAEF | 0.494 | 0.821 |
| Abnormal LAVi or Abnormal LAS_r_ or Abnormal LAEF | 0.924 | 0.231 |
| Abnormal LAVi + Abnormal LAS_r_ + Abnormal LAEF | 0.392 | 0.897 |

GLS indicates global longitudinal strain; LAVi, left atrial volume index; LAEF, LA emptying fraction; LAS_r,_ LA strain during reservoir phase.


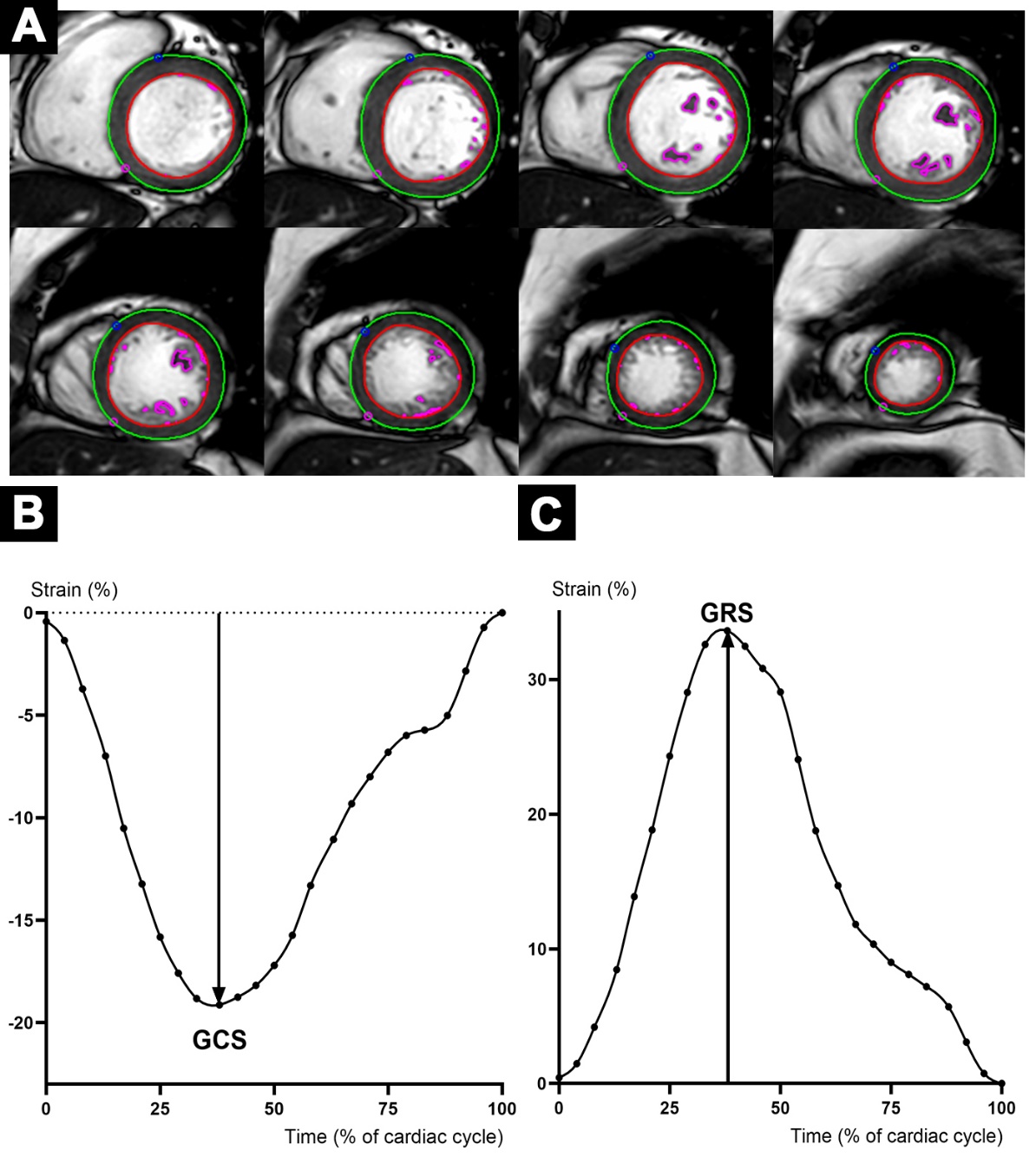


## **Figure S1.** Left ventricle contours demonstrated in short axis stack images **from cardiac magnetic resonance feature tracking (CMR-FT)**

Left ventricular (LV) endocardial (red line) and epicardial (green line) borders were semi-automatically drawn in each slice that had complete myocardial circumference with exclusion of papillary muscle and LV outflow tract. In-plane motion of each voxel point was tracked by the software for derivation of global circumferential strain (GCS) and global radial strain (GRS), each of which was reported as the peak value in its respective time-strain curve (B-C).


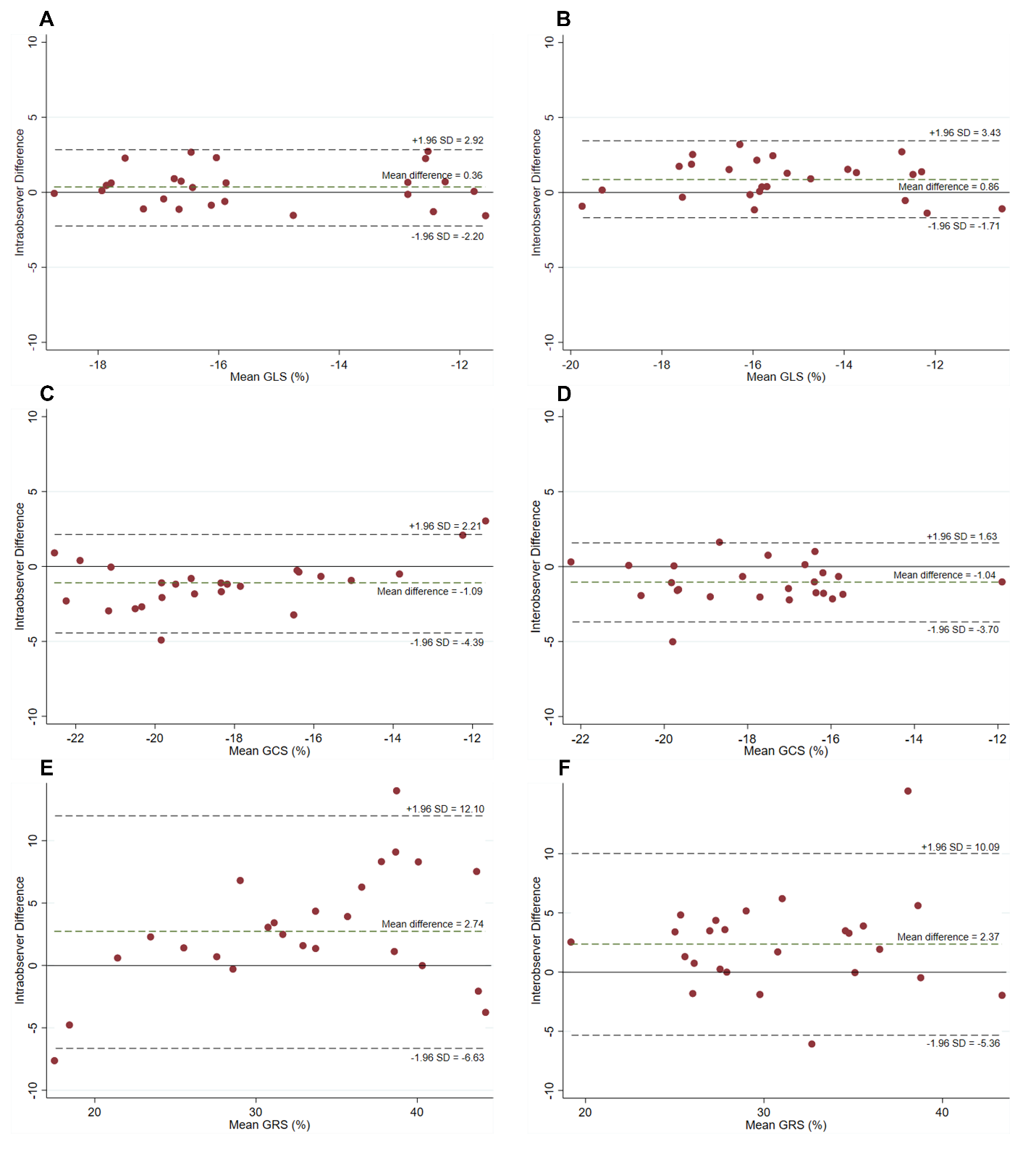


Figure S2. Bland Altman plots of LV strain parameters

Intraobserver and interobserver agreement of global longitudinal strain (GLS) (A and B), global circumferential strain (GCS) (C and D), and global radial strain (GRS) (E and F) are demonstrated. The upper and lower limits of agreement are shown as -1.96 SD and +1.96 SD of mean difference, respectively.


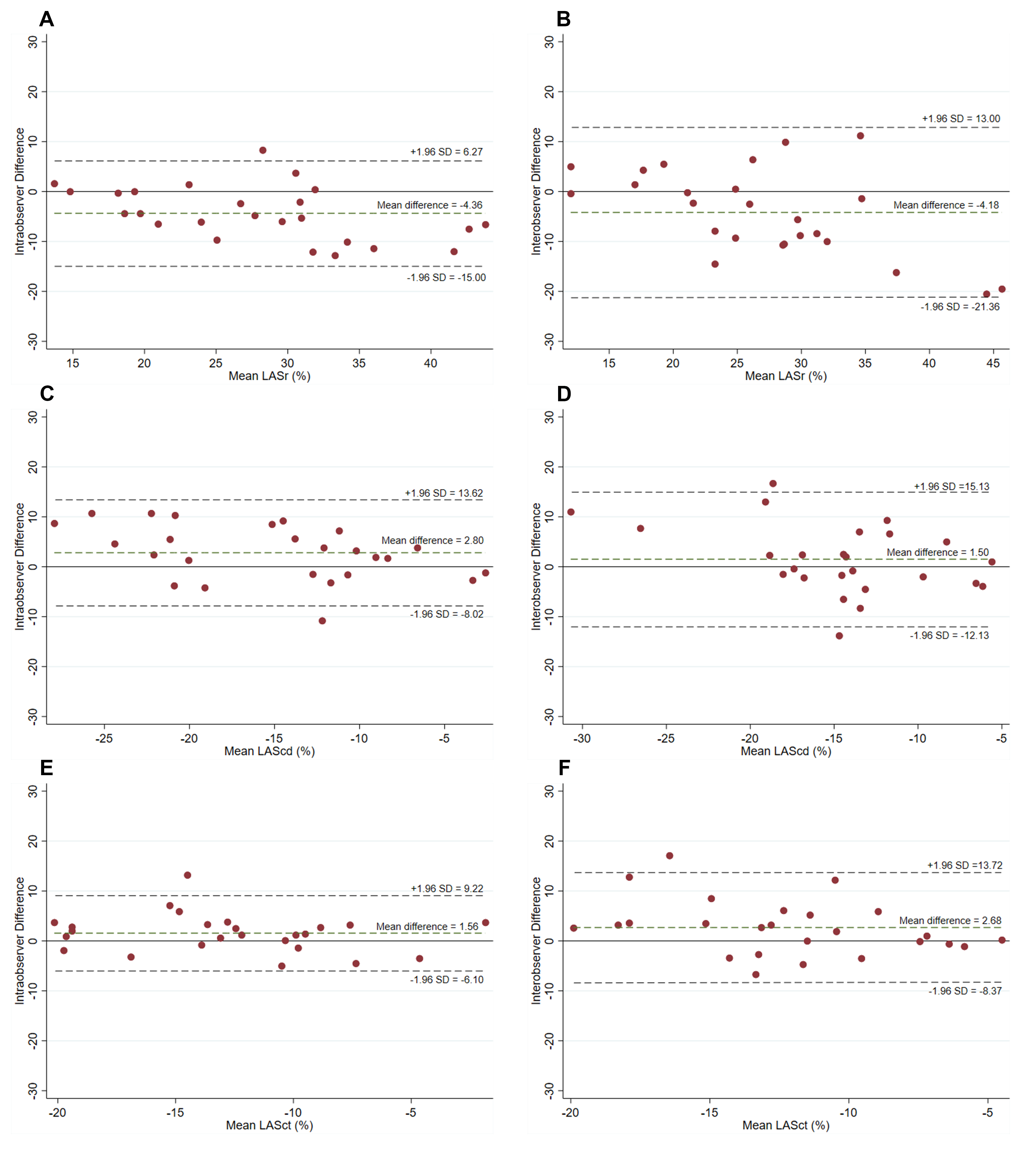


Figure S3. Bland Altman plots of LA strain parameters

Intraobserver and interobserver agreement of left atrial strain during reservoir phase (LAS_r_) (A and B), left atrial strain during conduit phase (LAS_cd_) (C and D), and left atrial strain during contraction phase (LAS_ct_) (E and F) are demonstrated. The upper and lower limits of agreement are shown as -1.96 SD and +1.96 SD of mean difference, respectively.

# Video Legends

Additional File 2: Video representing point tracking of LV in 2-chamber view

Additional File 3: Video representing point tracking of LV in 3-chamber view

Additional File 4: Video representing point tracking of LV in 4-chamber view

Additional File 5: Video representing point tracking of LA in 2-chamber view

Additional File 6: Video representing point tracking of LA in 4-chamber view
